# Graph complexity analysis identifies an *ETV5* tumor-specific network in human and murine low-grade glioma

**DOI:** 10.1371/journal.pone.0190001

**Published:** 2018-05-22

**Authors:** Yuan Pan, Christina Duron, Erin C. Bush, Yu Ma, Peter A. Sims, David H. Gutmann, Ami Radunskaya, Johanna Hardin

**Affiliations:** 1 Department of Neurology, Washington University School of Medicine, St. Louis, Missouri, United States of America; 2 Department of Mathematics, Claremont Graduate University, Claremont, California, United Strates of America; 3 Departments of Systems Biology and of Biochemistry & Molecular Biophysics, Columbia University Medical Center, New York, New York, United States of America; 4 Department of Mathematics, Pomona College, Claremont, California, United States of America; Northwestren University, UNITED STATES

## Abstract

Conventional differential expression analyses have been successfully employed to identify genes whose levels change across experimental conditions. One limitation of this approach is the inability to discover central regulators that control gene expression networks. In addition, while methods for identifying central nodes in a network are widely implemented, the bioinformatics validation process and the theoretical error estimates that reflect the uncertainty in each step of the analysis are rarely considered. Using the betweenness centrality measure, we identified *Etv5* as a potential tissue-level regulator in murine neurofibromatosis type 1 (*Nf1*) low-grade brain tumors (optic gliomas). As such, the expression of *Etv5* and *Etv5* target genes were increased in multiple independently-generated mouse optic glioma models relative to non-neoplastic (normal healthy) optic nerves, as well as in the cognate human tumors (pilocytic astrocytoma) relative to normal human brain. Importantly, differential *Etv5* and *Etv5* network expression was not directly the result of *Nf1* gene dysfunction in specific cell types, but rather reflects a property of the tumor as an aggregate tissue. Moreover, this differential *Etv5* expression was independently validated at the RNA and protein levels. Taken together, the combined use of network analysis, differential RNA expression findings, and experimental validation highlights the potential of the computational network approach to provide new insights into tumor biology.

## Introduction

Similar to other ecological systems, mammalian tissues can also be considered as complex biological systems, composed of a multitude of cellular and acellular elements that each contribute to overall biosystem function. In this regard, both normal and disease tissues contain numerous distinct cell types and molecular components that bi-directionally interact to establish new functional states for tumor tissue relative to their normal non-neoplastic counterparts. One natural outgrowth of this conceptualization is the idea that normal healthy and disease tissues can be defined in an objective manner using computational approaches. Algorithms have been used to classify diseased states, to assess risk as a function of specific factors such as gender, age, and environmental exposures [1–3]; and to identify individualized treatments based on gene expression profiles [4–6].

In addition to disease state classification, computational modeling can also be employed to identify ecosystem relationships that exist in the tissue as a whole. One type of model consists of representing relationships as a network, where the nodes are proteins, transcription factors, or genes, and the edges between nodes signify communication pathways. Networks highlight core differences between normal healthy and tumor tissues, and, as such, might serve to identify unanticipated regulatory pathways germane to the maintenance of the tumor. To investigate potential networks, we leveraged one authenticated murine model of a brain tumor (optic glioma) that arises in children with the neurofibromatosis type 1 (NF1) cancer predisposition syndrome [1, 2]. These optic glioma tumors are low-grade (World Health Organization grade I pilocytic astrocytomas) neoplasms, which develop early in childhood [7]. Since these tumors are not removed or biopsied in children with NF1, we specifically chose this *Nf1* genetically-engineered mouse low-grade glioma model system, because it recapitulates many of the features seen in the human condition and has been successfully employed to evaluate promising targeted therapies now in clinical trial for children with the tumors [3–6] (http://clinicaltrials.org; NCT01089101, NCT01158651 and NCT01734512).

Applications of network analysis to the understanding of cancer biology are relatively new, and can be divided into three methodological types: (1) enrichment of fixed gene sets, (2) *de novo* subnetwork construction and clustering and (3) network-based modeling [8]. While we applied all three methods to characterize murine *Nf1* optic gliomas, the network-based approach (the third type) was the only one with sufficient power to leverage relational network information to define specific regulator connections.

In this report, we describe one specific type of network analysis based on measures of network complexity [9]. We focused on a standard measure of network complexity predicated on the idea of centrality, as measured by *betweenness*. The general idea is that network complexity analysis reveals sub-networks that are the biggest contributors to genetic complexity. Genetic complexity equates to a “surplus of genotypic diversity over phenotypic diversity” [10], and is associated with networks that are more difficult to interpret. Our proof-of-principle study uses complexity analysis of weighted transcription networks to identify transcription factors that comprise a regulatory network unique to low-grade brain tumors arising in the optic nerves of *Nf1* mutant mice (optic gliomas). The edges of the transcription network are given weights based on transcription data, so that the RNA expression data from a normal control group result in a “normal” network, while data from a group of tumor samples are used to define a “tumor” network. Having a normal network and a tumor network side-by-side is important for comparing the two experimental conditions. Using both networks simultaneously, we were able to identify the Etv5 network as a defining feature of the neoplastic state in mouse and human low-grade glioma tumors.

## Materials and methods

### Mice

*Nf1*^*flox/flox*^ (N), *Nf1*^flox/null^; GFAP-Cre (OPG-1), *Nf1*^flox/R681^*; GFAP-Cre (OPG-2), *Nf1*^flox/null^; *Pten*^flox/wt^; GFAP-Cre (OPG-3), and *Nf1*^flox/null^; Olig2-Cre (OPG-4) mice [2, 11–13] were maintained on a C57Bl/6 background. Mice were housed in standard caging systems, which included irradiated commercial mouse chow and acidified water available at all times. Temperatures were kept at 70–72°F with 50–60% humidity on static racks. Light cycles were set at 5:00am on and 7:00pm off. All cages include pressed cotton squares for environmental enrichment that were changed multiple times per week. Every animal was checked at least once per day, and mating cages and cages with litters were checked twice per day for appearance, movement and overall health to monitor well-being. Mice were euthanized with 200 mg/kg sodium pentobarbital via i.p. prior to tissue isolation. All procedures were performed in accordance with an approved Animal Studies Committee protocol at Washington University with associated ethics committee approval.

### Immunohistochemistry

Optic nerves were microdissected and processed as described previously [14, 15]. Rabbit anti-Etv5 antibody (Novus Biologicals) was diluted at 1:100 for immunohistochemistry.

### RNA isolation

Mice were perfused, followed by optic nerves/chiasms microdissection, and preservation in TRIzol solution. RNA was isolated by phenol/chloroform extraction followed by isopropanol precipitation. RNA from primary astrocytes was isolated using the RNeasy Mini Kit (Qiagen) according to the manufacturer’s protocol.

### Quantitative real-time PCR

qPCR was performed as described previously [16]. The primers used for these experiments are listed in S1 Table.

### Primary astrocyte culture

Wild-type and *Nf1*-/- primary astrocytes were generated and maintained as previously described [17–20].

### RNA-seq experiments

RNA-sequencing of optic nerves/chiasms isolated from 6-weeks-old mice was performed as previously described [15].

### Regulatory network construction

The glioma network was inferred from the Rembrandt microarray data set (available from GEO GSE68848 [21]). The Rembrandt data were generated through the Glioma Molecular Diagnostic Initiative and include 874 glioma specimens. With the Rembrandt data, the network was built using the ARACNe-AP algorithm [22, 23].

The ARACNe-AP algorithm reconstructs gene regulatory networks from large-scale gene expression data [23]. The steps used in this algorithm are summarized below:

1. The input to the algorithm is a list of transcription facts and gene expression profile data.
2. The gene expression data is pre-processed in order to determine a mutual information (MI) threshold. Specifically, all pairwise MI scores between gene expression profiles are estimated, and then their significance assessed by comparing them to a null dataset. The significance level depends on the sample size of the input.
3. The bootstrapping step: selecting a random sample from the input gene expression profiles.
  - The gene expression profiles are rank-transformed, and then the MI for each Transcription Factor/Target pair is calculated.
  - The MI threshold from Step 2 is used to remove any connections that are not statistically significant.
  - Indirect interactions are removed using the Data Processing Inequality tolerance filter described in [22].
4. A network is constructed using consensus, by keeping only edges that were detected a significant number of times across all bootstrap runs in Step 3. Significance is determined using a Poisson distribution, keeping only those edges with a p-value less than 0.05.

The ARACNe algorithm, first described in [22], has been successfully used for over a decade. The Adaptive Paritioning version of the algorithm improves on the original method by eliminating the use of pre-determined bins of fixed sizes for estimating the mutual information. The resulting ARACNe-AP algorithm and its validation are described in detail in [23]. Various networks constructed using the algorithm are available online from bioconductor.org as the aracne.networks data package. The regulatory network used in this study is provided as part of the supplementary materials (S2 Table).

### Centrality calculations

RNA expression data from optic nerves of *Nf1*^flox/flox^ (N, normal control group, *n* = 4) and *Nf1*^flox/mut^; GFAP-Cre (OPG-1, tumor group, *n* = 11) were generated previously [15] and used to create separate weighted networks. Note that the network structure is the same for the two groups, but the weights assigned to the edges differ. Genes that were not expressed in one of the groups were removed from the network. The network weights are given by the distance between two nodes (i.e., between two genes). The distances between two genes (*j* and *k*) are calculated as 1 − | *ρ*(*x*_*j*_, *x*_*k*_)| where *ρ*(*x*_*j*_, *x*_*k*_) is the minimum of the Pearson and Spearman correlations between the expression levels of gene *j* and gene *k*.

The betweenness centrality of gene *i* is the fraction of shortest paths in the network that go through gene *i*. Note that our original choice of transcriptome network built by the ARACNe-AP algorithm has a consequence that only regulators (and not their targets) have high centrality measures. Centrality measures were calculated using the *igraph* package in R [24].

### Differential expression analysis

The differential expression and fold change calculations on the RNA-Seq datasets (including all of the OPG datasets) were performed using the R package *DESeq2* version 1.18.1 [25], including use of Benjamini-Hochberg adjusted p-values [26] with an adjusted significance level of 0.01. Published microarray data were analyzed by comparing pilocytic astrocytomas and normal control samples. The publicly available data were already normalized, and we performed standard two-sample t-tests on all probes in each dataset. After analysis at the probe level, data were collated based on the gene to which the probes mapped. Because the analysis was confirmatory for ETV5 and its network (and not exploratory), we did not adjust for multiple comparisons.

### Details of the datasets employed

**Fig 1:**
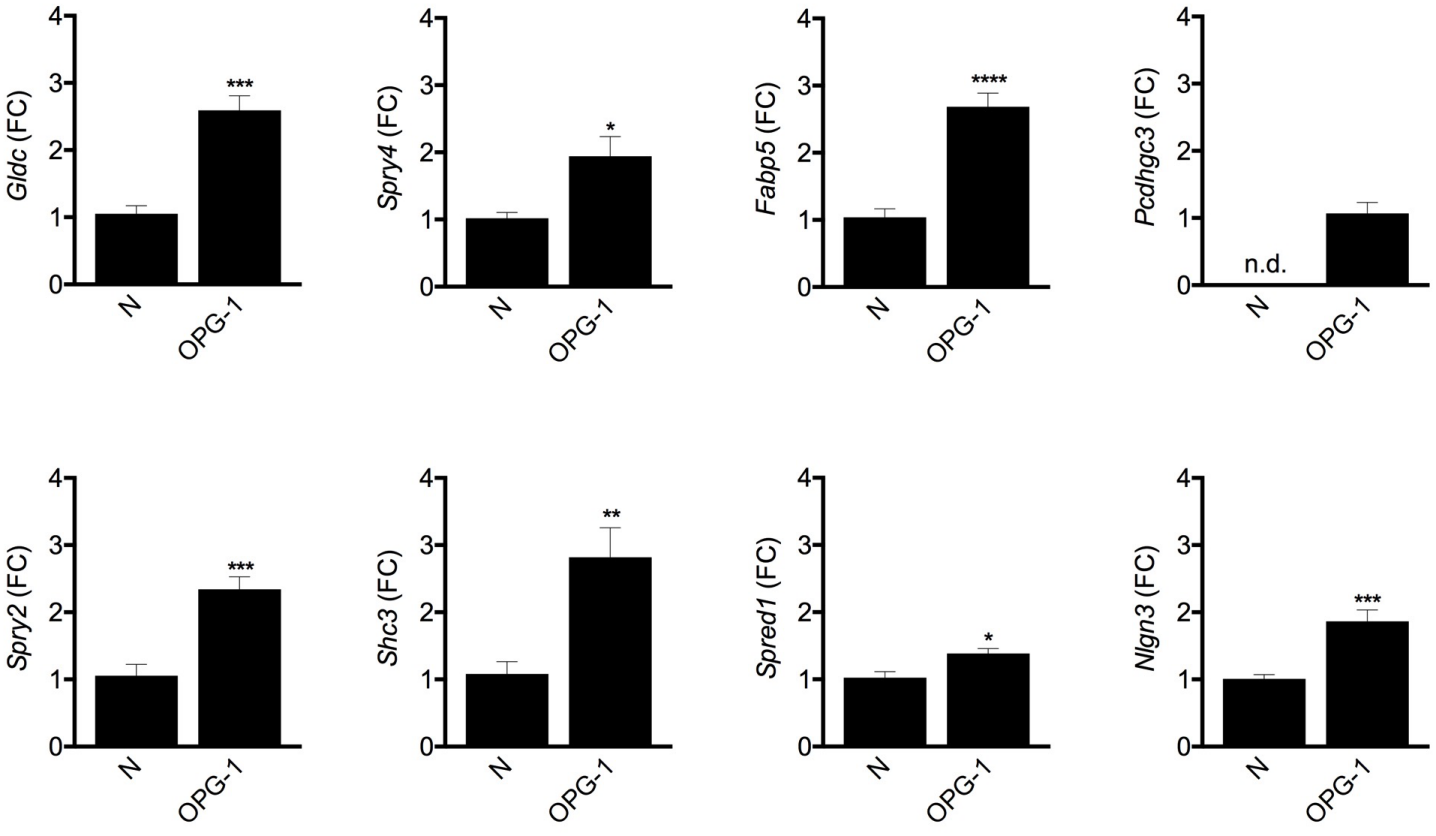
The *Etv5* network is differentially expressed in murine *Nf1* optic gliomas. *Gldc*, *Spry4*, *Fabp5*, *Pcdhgc3*, *Spry2*, *Shc3*, *Spred1* and *Nlgn3* RNA expression is increased in optic glioma samples (OPG-1) relative to the normal murine optic nerve (N). n.d., not detected. FC, fold change. p = 0.0003 (*Gldc*), p = 0.0258 (*Spry4*), p<0.0001 (*Fabp5*), p = 0.0005 (*Spry2*), p = 0.0045 (*Shc3*), p = 0.0111 (*Spred1*), p = 0.0008 (*Nlgn3*). The unadjusted p-values were calculated separately using a t-test.

**Fig 2:**
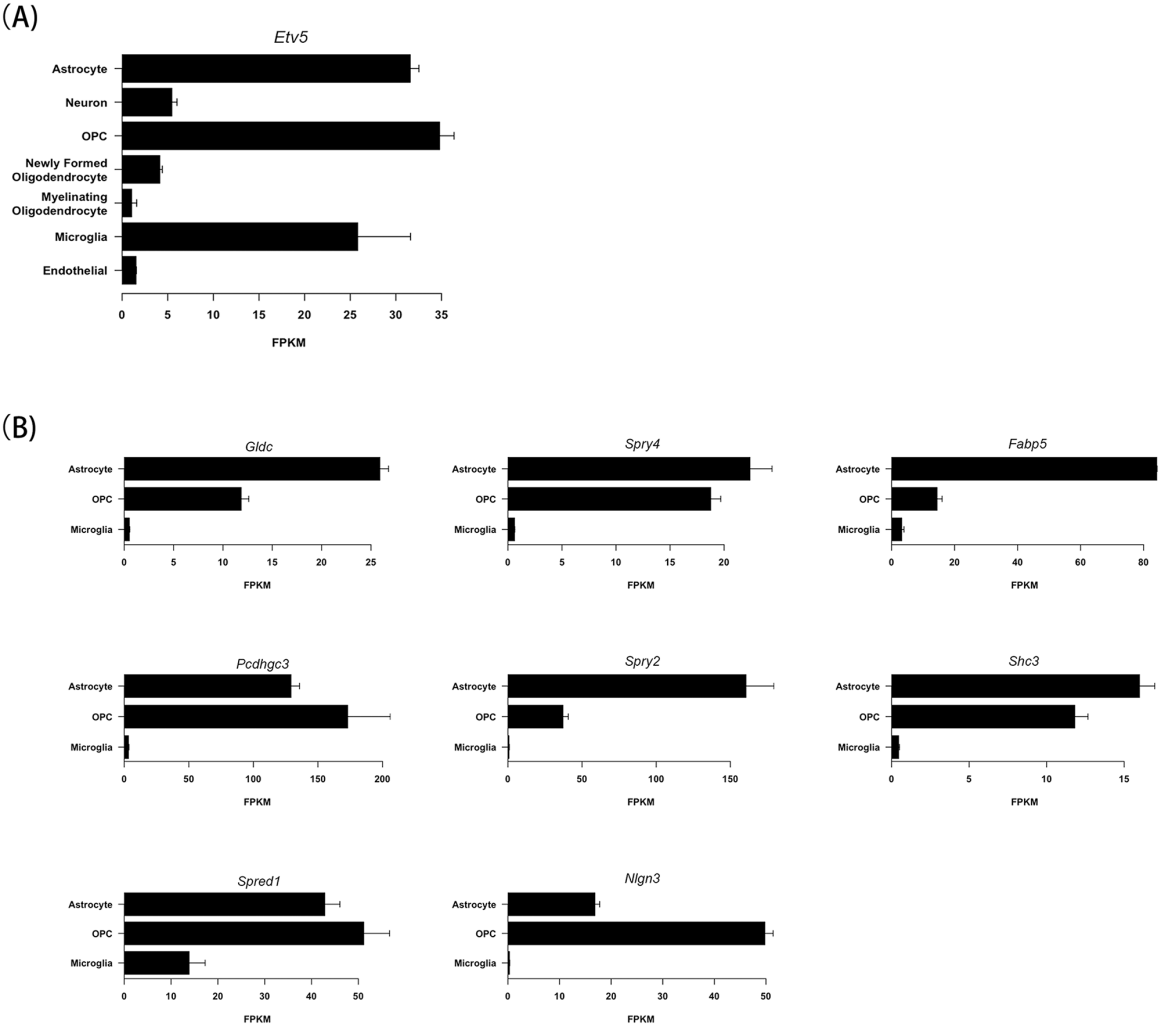
Expression of Etv5 network in the central nervous system. **(A)** *Etv5* RNA expression in astrocytes, neurons, OPCs (oligodendrocyte progenitor cells), newly formed oligodendrocytes, myelinating oligodendrocytes, microglia and endothelial cells demonstrates preferential enrichment in astrocytes, OPCs and microglia. Data analyzed from the brain RNA-Seq database [27] (http://web.stanford.edu/group/barres_lab/brain_rnaseq.html). **(B)** *Gldc*, *Spry4*, *Fabp5*, *Pcdhgc3*, *Spry2*, *Shc3*, *Spred1* and *Nlgn3* expression was enriched in astrocytes, OPCs and microglia. *Spred1* was the only gene expressed in microglia.

1. RNA-Seq Data for 3-month-old murine non-neoplastic and OPG-1, OPG-2, OPG-3, OPG-4 models. RNA-Seq murine data that formed the basis of the original and follow-up analyses. The non-neoplastic samples are also denoted as FF (*Nf1*^flox/flox^); OPG-1 model is also denoted as FMC (*Nf1*^flox/null^; GFAP-Cre); the OPG-2 model is also denoted as F18C (*Nf1*^flox/R681^*; GFAP-Cre); the OPG-3 is also denoted as FMPC (*Nf1*^flox/null^; *Pten*^flox/wt^; GFAP-Cre); and the OPG-4 model is denoted as FMOC (*Nf1*^flox/null^; Olig2-Cre). The samples are publicly available at https://www.ncbi.nlm.nih.gov/geo/query/acc.cgi?acc=GSE102345.
2. RNA-Seq data for 6-week-old murine non-neoplastic and *Nf1* OPG-1 model0 RNA-Seq murine data on 6-week-old OPG-1 (FMC) mice. The OPG-1 model is also denoted as FMC. The samples are publicly available at https://www.ncbi.nlm.nih.gov/geo/query/acc.cgi?acc=GSE102345.
3. qPCR data for 3-month-old murine *Nf1* OPG-1 model. In these experiments, we chose 12 genes (*Gldc*, *Spry4*, *Fabp5*, *Pcdhgc3*, *Spry2*, *Shc3*, *Spred1*, *Nlgn3*, *Dusp6*, *Lrp4*, *Rsbn1l*, and *Socs2*) for qPCR validation in optic nerves from normal healthy and optic glioma-bearing mice (n = 3 mice/group), 8 genes that demonstrated differential expression patterns similar to the original RNA-seq data (*Gldc*, *Spry4*, *Fabp5*, *Pcdhgc3*, *Spry2*, *Shc3*, *Spred1* and *Nlgn3*, Fig 1), while 4 demonstrated different patterns or no change (*Dusp6*, *Lrp4*, *Rsbn1l* and *Socs2*, data not shown). The p-values were calculated using a t-test (GraphPad, Prism).
4. Cell-type based expression data from the brain RNA-Seq database. Data analyzed from the brain RNA-seq database [27], Fig 2 compares the expression levels in various cell types. The samples are publicly available at http://web.stanford.edu/group/barres_lab/brain_rnaseq.html.
5. Microarray data for pediatric pilocytic astrocytomas. The dataset is available at the NCBI GEO repository, GSE42656 (https://www.ncbi.nlm.nih.gov/geo/query/acc.cgi?acc=GSE42656), which included 14 pediatric pilocytic astrocytomas and 8 fetal cerebellum samples. The gene expression samples were measured using Illumina HumanHT-12 V3.0 expression beadchip [28]. In addition, GSE12907 (https://www.ncbi.nlm.nih.gov/geo/query/acc.cgi?acc=GSE12907), contained 21 juvenile pilocytic astrocytomas and four normal cerebellum samples. The gene expression samples were measured using an Affymetrix Human Genome U133A array [29].

## Results

The network complexity analysis in this case study is divided into two steps: (1) discovery of *Etv5* with computational methods and (2) validation of *Etv5* with independent experimental techniques. The edges of the transcription network are given weights based on expression data such that expression data from a normal control group (N, optic nerves from *Nf1*^flox/flox^ mice, which are equivalent to wild-type mice [2]), result in a “normal” network, while data from a group of optic glioma tumor samples (OPG-1, optic nerves from *Nf1*^flox/null^; GFAP-Cre mice, [2]) comprise a “tumor” network. While the individual bioinformatics steps are not new, our novel approach illustrates how this practical approach can be implemented to identify unknown regulatory networks.

First, we use network complexity to identify genes that are central to the tumor network. Herein, the term central is used in the context of network complexity analysis: a node’s centrality measure is determined by the frequency with which it appears in paths between other nodes in the network, as described below. Next, we identify a single transcription factor, *Etv5*, not previously implicated in low-grade glioma, as the gene most **differentially central**: As such, *Etv5* was the gene most central in the tumor network, and was not central in the normal network. Finally, we validated the differential expression of *Etv5* in several other *Nf1* murine models of optic glioma, as well as in human low-grade glioma (pilocytic astrocytoma, PA) datasets. This validation was accomplished by comparing expression levels of *Etv5* and its target genes in normal healthy optic nerves and optic gliomas, as well as by determining the cell type expression profiles of those genes.

### Bioinformatic discovery of Etv5

In our analysis, we employed two distinct computational techniques (network and differential expression analyses) to provide different information about the relationships between the genes within the networks and across the experimental conditions. The discovery of *Etv5* and its network was only possible using the combination of the different computational techniques (see Algorithm 1).

### Network and complexity analysis

In order to identify genes that might play a role in disease evolution, we sought to determine what has changed in the intra-cellular network that describes cellular processes. We also sought to identify genes whose role had significantly changed as the tissue evolved from a healthy to a diseased state. This study describes how to combine a centrality analysis with differential expression to identify potential genes of interest. In order to do this, we required a network that represented possible interactions between genes. We utilized expression data from samples in different groups (healthy and diseased, for example) to add weights to an existing interactome to create a weighted network for each group. This allowed us to identify “important” subnetworks in each network, and to determine which genes are central to the important subnetworks in the diseased tissue.

A gene regulatory network (GRN) depicts how some genes encoding regulatory molecules, such as transcription factors or microRNAs, regulate the expression of other genes [30]. As the base network for the *Etv5* analysis, we used a regulatory network based on biological interactions between transcription factors and their targets. This network was developed in prior research by another group, and is described more fully in [23]. The nodes of the network represent regulator and target genes, and the edges represent protein-DNA interactions. The regulatory network was derived from transcription (RNA) data, representing a variety of different gliomas, including low-grade (benign) and high-grade (malignant) gliomas, to capture pan-glioma regulatory interactions. Four hundred twenty-seven human glioma gene expression profiles were obtained from the Rembrandt data repository [21], and these were combined to create a network using the ARACNe-AP (Algorithm for the Reconstruction of Accurate Cellular Networks). This network was selected as the most appropriate to use, since it is based on transcription data from gliomas. We added edge weights to this network using RNA expression data, as described below. The details of the ARACNe algorithm are provided in [23]: The basic procedure uses mutual information (MI) to measure similarity across genes and then bootstrapping to measure the strength and consistency of the similarities. A step-by-step description of the algorithm is presented in the Materials and Methods section. The regulatory network is available as S2 Table.

**Fig 3:**
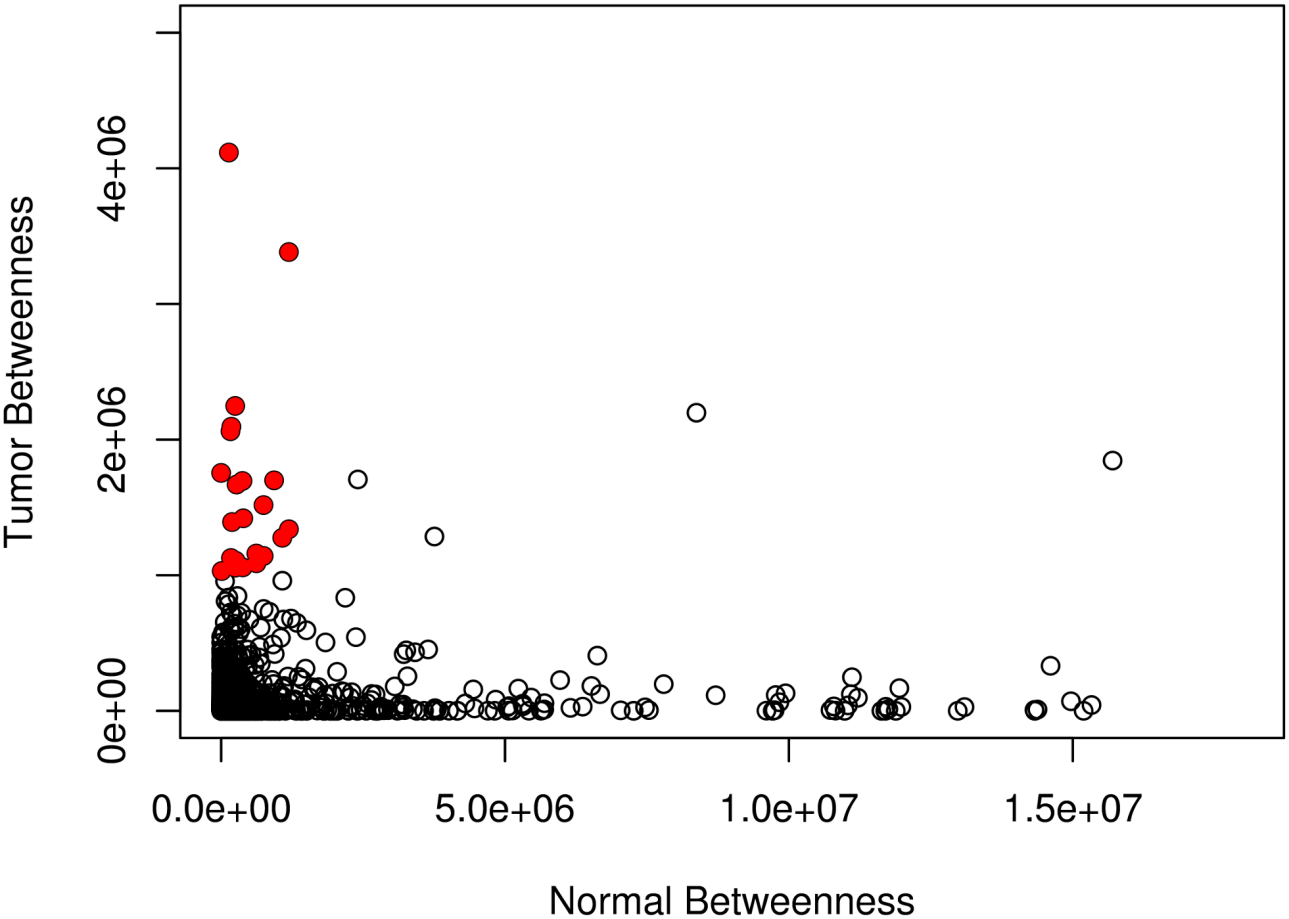
Comparison of betweenness measures in the normal and tumor networks. Filled (red) circles indicate genes whose betweenness measure is at least 1.1 times as large in the tumor network as in the normal network and either a tumor betweenness or normal betweenness value greater than 1e6. These genes are listed in Table 1, and shown in pink in Fig 4.

Centrality measures are commonly used to identify nodes that could potentially play important roles in weighted networks [31]. Some frequently used centrality measures are closeness, betweenness, and entropy; for example West et al. use differential entropy between tumor and normal tissue to detect relevant genes [32]. The betweenness measure was selected as the best for distinguishing between normal control and tumor-weighted networks, since its large range of values led to a clear discrimination between the tumor and control networks. Fig 3 and S1 Fig show the comparison of using betweenness and closeness to differentiate the tumor and normal networks. The betweenness metric identifies genes that are substantially different across the two networks; the closeness metric does not identify any such stand-out genes. It should be noted that *Etv5* was not among the 50 most differentially-expressed genes by standard differential expression analysis, and it is unlikely that it would have been identified as a potential glioma network regulator.

The *Betweenness Centrality* of gene *i* (i.e., node *i*), *b*_*i*_, is the fraction of shortest paths in the network that go through gene *i*:

$$b_{i}\;=\;\underset{i\neq j\neq k}{\sum }\frac{n_{j,k}(i)}{n_{j,k}}$$

where *n*_*j*,*k*_ is the number of shortest paths connecting genes *j* and *k*, and *n*_*j*,*k*_(*i*) is the number of shortest paths that also go through gene *i*. The length of the path between any two genes is given by the sum of the distances, or weights, of its edges, where the distances were computed using the expression data. Because the edge values are based on the expression data, the betweenness measures will differ between the normal and tumor expression networks. Importantly, the genes with larger betweenness values in the tumor expression network relative to the normal expression network were identified.

Using the 3-month-old murine OPG-1 RNA-Seq dataset, 22 genes were identified as having betweenness values at least 1.1 times as large in the tumor expression network as in the normal expression network and either a tumor betweenness or normal betweenness value greater than 1e6. These genes are shown as solid, red circles in Fig 3; listed in Table 1; and denoted as “central” to the expression network.

**Table 1:** “Central genes”: Transcription factors identified as having betweenness values at least 1.1 times as large in the tumor expression network as in the normal expression network and either a tumor betweenness or normal betweenness value greater than 1e6.

*Cebpz*	*Etv5*	*Spen*	*Zcchc14*	*Camta1*	*Chd5*	*Cers2*
*Hnrnpab*	*Ilf2*	*Zcchc17*	*Zc3h15*	*Tulp4*	*Purb*	*Rpl7*
*Tcf3*	*Tead1*	*Cnbp*	*Prdm2*	*Sarnp*	*Zranb2*	*Gcsh*
*Ift74*	*Myl12B*					

### Algorithm 1: Identifying *Etv5* network

1. A reference network is identified for all transcription factors. In this study we use the regulatory network created with the ARACNe-AP algorithm
2. Weights are added to the edges of the reference network using one minus the minimum of the Pearson or Spearman correlation of RNA-Seq data. Two different weighted networks are created—one with tumor RNA-Seq data and one with normal RNA-Seq data
3. From each weighted network, the betweenness value for each gene is calculated
4. Genes with betweenness values 1.1 times as big in the tumor network relative to the normal network *and* having either a tumor betweenness or a normal betweenness greater than 1e6 are identified. These genes are considered “central” in the tumor network (Table 1).
5. Of the “central” genes, the most differentially expressed gene is identified (*Etv5*).
6. Genes which represent targets of the *Etv5* transcription factor **and** are differentially expressed across the two conditions were identified (Table 2).

**Table 2:** Thirty-one targets of *Etv5* are differentially expressed in 3-month-old murine OPG-1 RNA-Seq samples.

GENE	p-value	Adjusted p-value	log2 Fold Change
*Spry2*	1.13E-08	2.41E-06	0.916
*Dnajb4*	2.63E-05	1.65E-03	-0.457
*Col2a1*	1.07E-04	4.43E-03	1.235
*Spred1*	1.04E-05	7.78E-04	0.800
*Dusp6*	1.50E-04	5.54E-03	0.637
*S1pr1*	6.84E-17	9.28E-14	1.358
*Ak4*	5.38E-05	2.77E-03	0.959
*Fabp5*	1.39E-09	3.93E-07	1.087
*Fabp7*	6.22E-05	3.02E-03	0.956
*Rsbn1l*	2.16E-04	7.15E-03	-0.428
*Btbd3*	7.12E-09	1.62E-06	0.755
*Gap43*	1.94E-05	1.27E-03	-0.797
*Gja1*	3.64E-10	1.26E-07	0.600
*Gldc*	6.60E-06	5.29E-04	1.161
*Kcnip1*	3.06E-04	9.09E-03	-0.797
*Igfbp6*	3.07E-04	9.10E-03	-0.942
*Lrp4*	5.38E-05	2.77E-03	0.671
*Mmp15*	2.11E-04	7.07E-03	1.003
*Nt5e*	1.18E-04	4.76E-03	-0.744
*Pcdhgc3*	2.12E-06	2.09E-04	0.932
*Tppp3*	6.01E-05	2.94E-03	-0.912
*Shc3*	2.71E-04	8.35E-03	0.903
*Nlgn3*	1.82E-05	1.21E-03	0.705
*Spata6*	1.62E-04	5.86E-03	-0.552
*Elovl2*	1.62E-11	9.28E-09	1.908
*Spry4*	5.77E-05	2.87E-03	1.104
*Socs2*	1.77E-04	6.28E-03	-0.814
*Slc9a3r1*	1.41E-06	1.51E-04	0.722
*Chst2*	2.28E-11	1.26E-08	1.163
*Cxcl14*	4.88E-17	7.29E-14	1.425
*Dock4*	2.88E-05	1.73E-03	0.642

### Identification of ETV5

Once the twenty-three “central” genes were identified, we determined which of them behaved differently in the two groups with respect to average RNA expression. One gene, *Etv5*, emerged as having a large differential effect across the two expression datasets on the 3-month-old murine OPG-1 samples. Using the original regulatory network, we identified the target genes for each of the regulators listed in Table 1. Differential expression analysis was employed to identify which of the central genes and which of their target genes were significantly different between the tumor (OPG-1) and the normal (N) 3-month-old samples.

**Fig 4:**
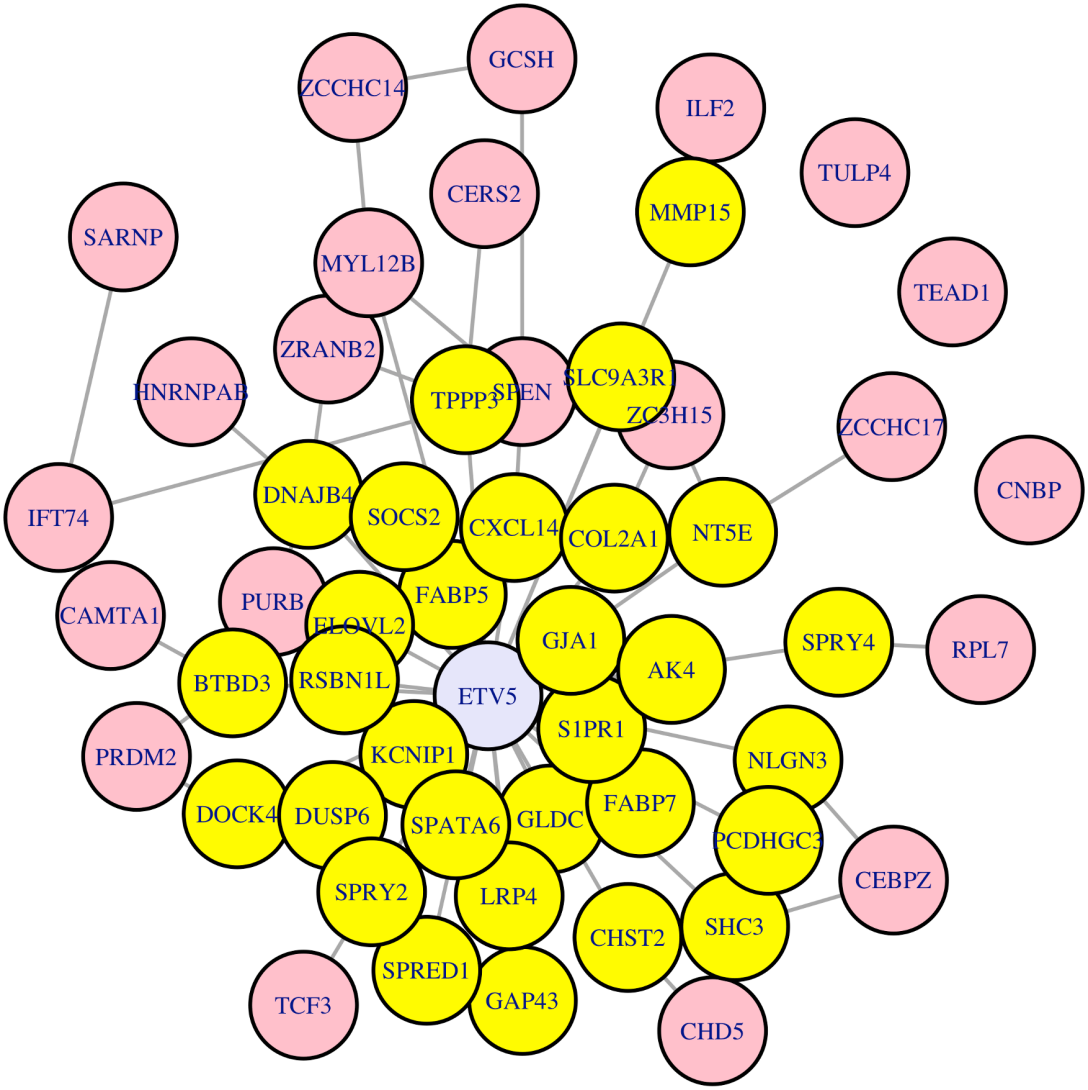
The *Etv5* network. The subnetwork is comprised of *Etv5* (in lavender in the center), and its differentially-expressed targets (shown in yellow). The remaining central genes, identified by their high betweenness measures relative to the normal network, are shown on the periphery in pink.

Importantly, of the regulator genes that were determined to be “central” according to the betweenness measure (Table 1), the gene that was mostly clearly differentially expressed was *Etv5* (Table 3). Additionally, *Etv5* also had the highest percentage of differentially-expressed targets of all of the central genes (Table 4). A list of the 31 differentially-expressed *Etv5* target genes is shown in Table 2. The network consisting of the central regulators, along with the differentially expressed targets of *Etv5* is shown in Fig 4. Henceforth, we call the subnetwork consisting of *Etv5* and its differentially expressed targets as “the Etv5 network”.

**Table 3:** Differential expression of central genes identified using the betweenness measure.

GENE	p-value	Adjusted p-value	log2 Fold Change
*Etv5*	1.34E-09	3.87E-07	1.4474
*Myl12B*	6.81E-04	1.61E-02	-0.5850
*Zc3h15*	4.03E-03	5.12E-02	-0.5603
*Camta1*	4.22E-03	5.24E-02	-0.4705
*Spen*	4.61E-03	5.55E-02	0.5714
*Tulp4*	6.11E-03	6.48E-02	0.4845
*Sarnp*	7.36E-03	7.24E-02	0.7022
*Zcchc17*	9.34E-03	8.26E-02	-0.6699
*Ift74*	9.66E-03	8.43E-02	-0.5475
*Tcf3*	1.29E-02	9.89E-02	0.4928
*Zcchc14*	3.82E-02	1.80E-01	0.3660
*Rpl7*	3.83E-02	1.80E-01	-0.5100
*Cnbp*	4.16E-02	1.88E-01	-0.3724
*Zranb2*	6.71E-02	2.47E-01	-0.3515
*Tead1*	1.40E-01	3.70E-01	0.2794
*Ilf2*	1.51E-01	3.83E-01	-0.3077
*Cebpz*	1.60E-01	3.96E-01	-0.3255
*Gcsh*	3.92E-01	6.38E-01	-0.1581
*Cers2*	5.36E-01	7.49E-01	-0.0923
*Hnrnpab*	6.23E-01	8.06E-01	0.0784
*Purb*	6.80E-01	8.43E-01	0.0573
*Prdm2*	8.75E-01	9.45E-01	0.0207
*Chd5*	8.98E-01	9.57E-01	-0.0334

**Table 4:** Percent of target genes (from the regulatory network) that are differentially expressed by each of the central genes identified using the betweenness measure.

GENE	#significant	Total targets	% significant
*Etv5*	31	449	0.0690
*Cers2*	32	496	0.0645
*Sarnp*	15	255	0.0588
*Zcchc14*	16	361	0.0433
*Tcf3*	28	640	0.0437
*Tead1*	19	443	0.0429
*Zc3h15*	27	652	0.0414
*Tulp4*	17	412	0.0413
*Rpl7*	10	247	0.0405
*Purb*	27	678	0.0398
*All*	529	14926	0.0354
*Hnrnpab*	19	543	0.0350
*Cnbp*	15	458	0.0328
*Camta1*	35	1093	0.0320
*Chd5*	27	852	0.0317
*Zcchc17*	8	257	0.0311
*Prdm2*	20	652	0.0307
*Spen*	12	392	0.0306
*Cebpz*	10	379	0.0264
*Ilf2*	13	555	0.0234
*Zranb2*	11	486	0.0226
*Gcsh*	0	0	0
*Ift74*	0	0	0
*Myl12B*	0	0	0

### Experimental validation

To extend the *in silico* findings, and to validate *Etv5* as a differentially-expressed tumor-specific gene, we performed a series of complementary experiments. These experiments showed that *Etv5* and its targets are differentially expressed in different cell types, at different stages of tumor growth, and in different organisms, and lend further credence to the notion that *Etv5* and its associated target genes comprise a potential regulatory network in neoplastic tissue relative to their normal healthy, non-neoplastic counterparts.

**Fig 5:**
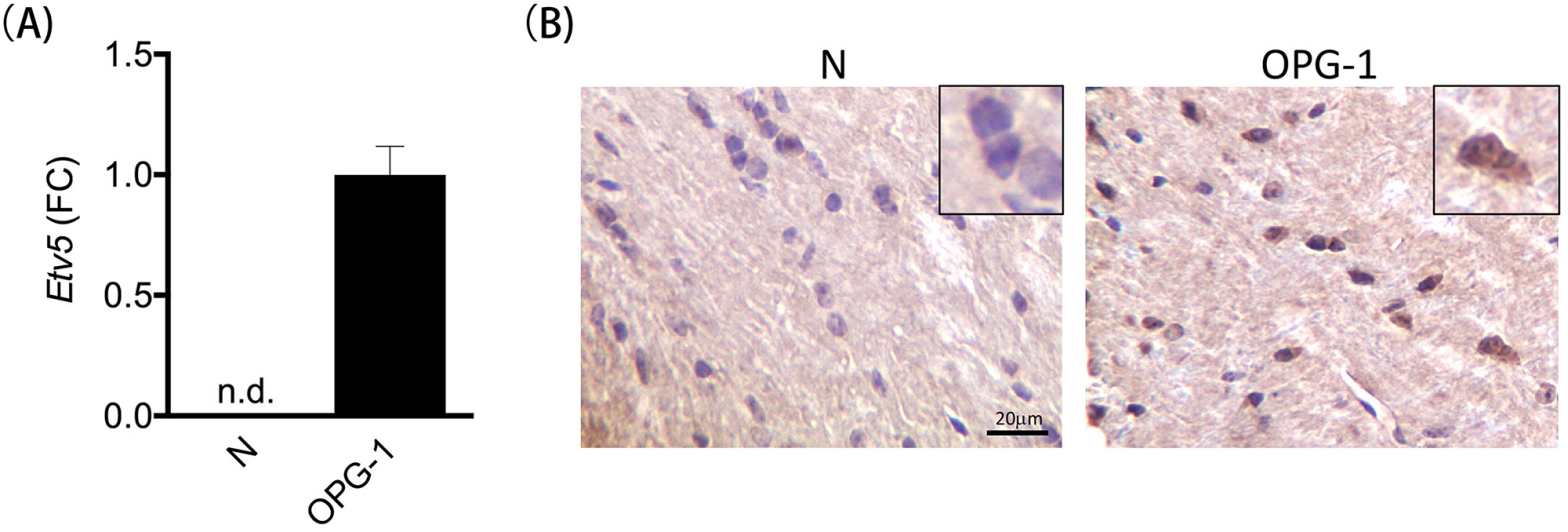
*Etv5* differential expression is confirmed using qPCR on independently-generated murine *Nf1* optic gliomas. **(A)** *Etv5* expression was detected in in one representative murine *Nf1* optic glioma model (OPG-1) but was not detected (n.d.) in the normal murine optic nerve (N) by qPCR. **(B)** Immunohistochemistry using Etv5-specific antibodies demonstrates Etv5 protein expression in the optic glioma (OPG-1, shown in brown), but not in the normal optic nerve (N). Nuclei are stained with hematoxylin (shown in blue). Scale bar, 20μm.

In addition to RNA-Seq experiments, we also used quantitative real-time PCR (qPCR) to analyze *Etv5* RNA expression in independently-generated non-neoplastic (*Nf1*^flox/flox^ [2]; abbreviated N for normal) and tumor-bearing (*Nf1*^flox/null^ GFAP-Cre [2]; “OPG-1”) optic nerves. For these studies, four nerves and chiasms from each group were removed from 3-month-old mice at a time when optic gliomas in these mice are first detected [33]. Consistent with the RNA-seq data, *Etv5* expression was detected in the tumor-bearing optic nerves, but not in their non-neoplastic counterparts (Fig 5A). Moreover, Etv5 protein expression was also observed in optic glioma tissue, but not in normal healthy optic nerves, by immunohistochemistry with an Etv5-specific antibody on paraffin-embedded formalin-fixed specimens (Fig 5B). Collectively, these results validate the tumor-specific expression of Etv5 at both the RNA and protein levels.

As an additional method to strengthen the specificity of the observed differential *Etv5* expression in the neoplastic tissue, we performed RNA-seq analysis on 6-week-old optic nerves from normal healthy and optic glioma-bearing mice (n = 3 mice/group). At this stage of glioma development, the tumors are just beginning to form, as evidenced by an increase in tumor proliferation and glial cell numbers, but without a change in optic nerve volume [33]. Similar to the results obtained from 3-month-old mouse specimens, there was differential *Etv5* RNA-Seq expression in 6-week-old optic glioma-bearing mice, which contain a developing, but not mature, tumor [33] (S2 Fig). These observations suggest that differential expression of *Etv5* defines a ground state of neoplasia that exists at a time when it is not yet possible to clearly classify the mouse optic nerve as a tumor.

Numerous prior studies from our laboratory have employed differential RNA expression methods to identify genes unique to murine *Nf1* optic glioma. These include an analysis of neoplastic and non-neoplastic cell types; however, none of these previous studies identified *Etv5* as a differentially-expressed gene [16, 34]. In this respect, previous studies including gene expression profiles for isolated cells in optic glioma did not find that *Etv5* was differentially regulated in any specific single cell type (*e*.*g*., astrocytes, microglia). These particular findings were confirmed by the analysis of *Nf1*-deficient neoplastic cells (astrocytes) by qPCR (S3 Fig) and tumor-associated microglia (non-neoplastic cells [16]) using isolated cells and data previously acquired. Taken together, these observations argue that differential *Etv5* expression reflects a “whole tumor” property, rather than representing the individual contributions from any single cell type within the tumor.

Next, we sought to determine whether the target genes of *Etv5*, which were differentially expressed in the tumor, are similarly differentially expressed in tumor-bearing optic nerves relative to normal healthy non-neoplastic optic nerve. In these experiments, we chose 12 genes (*Gldc*, *Spry4*, *Fabp5*, *Pcdhgc3*, *Spry2*, *Shc3*, *Spred1*, *Nlgn3*, *Dusp6*, *Lrp4*, *Rsbn1l*, and *Socs2*) for independent validation by qPCR (Fig 1). Of the 12 genes chosen, 67% demonstrated differential expression patterns similar to the original RNA-seq data (*Gldc*, *Spry4*, *Fabp5*, *Pcdhgc3*, *Spry2*, *Shc3*, *Spred1* and *Nlgn3*), while four demonstrated different patterns or no change (*Dusp6*, *Lrp4*, *Rsbn1l* and *Socs2*).

Because the expression of Etv5 targets in the tumor may reflect the expression of these genes in different cell types, we queried an established brain cell type-specific transcriptome database [27]. We focused on *Etv5* and those targets that were significantly differentially expressed (*Gldc*, *Spry4*, *Fabp5*, *Pcdhgc3*, *Spry2*, *Shc3*, *Spred1* and *Nlgn3*). Using this brain RNA-Seq database [27], we found enrichment of *Etv5* expression in astrocytes, oligodendrocyte progenitor cells (OPC) and microglia relative to endothelial cells, neurons, and more differentiated oligodendrocytes (Fig 2A). These findings are consistent with biological studies demonstrating that astrocytes [2], o-GSCs (optic glioma stem cells) [17], and microglia [6, 16] are all critical mediators of glioma formation and growth. Interestingly, seven of the eight *Etv5* target genes were expressed predominantly in o-GSCs and astrocytes, whereas *Spred1* was expressed in all three cell types (Fig 2B). These observations additionally support the idea that the network formed by *Etv5* and its targets reflects a global change in the tumor ecosystem, rather than differential gene expression by only one cell type in the tumor.

To demonstrate that differential *Etv5* expression is a hallmark of murine *Nf1* optic glioma, we leveraged prior data from our laboratory in which multiple distinct genetically-engineered mouse models of optic glioma were generated. Each of these models differs in important respects, including the germline *Nf1* gene mutation (OPG-2; c.2041C>T; p.R681X as seen in several patients with NF1-OPG [12]), the presence of additional genetic mutations (OPG-3; additionally harboring heterozygous *Pten* loss [11]), and the tumor cell of origin (OPG-4; *Nf1*^flox/mut^; Olig2-Cre in which tumors arise from Olig2^+^ cells). While the four optic glioma models are histologically similar to each other, they represent molecularly-distinct tumors, as assessed by RNA-seq analysis [15]. In each of the murine *Nf1* OPG models, we observed increased *Etv5* RNA expression (Table 5). Moreover, in at least three out of the four optic glioma models, there is increased expression of the eight validated *Etv5* target genes (*Gldc*, *Spry4*, *Fabp5*, *Pcdhgc3*, *Spry2*, *Shc3*, *Spred1* and *Nlgn3*) (Table 5). These findings further strengthen the conclusion that differential *Etv5* expression is a hallmark of the neoplastic state, as it is shared amongst multiple distinct murine *Nf1* optic glioma models.

**Table 5:** Summary of differential *Etv5* expression in additional murine *Nf1* optic glioma models.

Gene	OPG-1	OPG-2	OPG-3	OPG-4
*Etv5*	2.74	3.54	2.40	2.07
*Gldc*	2.25	2.85	1.76	1.50
*Spry4*	2.16	3.04	1.81	1.81
*Fabp5*	2.13	n.s.	1.69	2.15
*Pcdhgc3*	1.91	1.91	1.65	1.44
*Spry2*	1.89	1.62	1.37	n.s.
*Shc3*	1.88	2.45	1.74	1.84
*Spred1*	1.74	1.96	n.s.	1.71
*Nlgn3*	1.63	1.74	1.61	1.62

Values connote fold changes in optic glioma (OPG) RNA expression relative to non-neoplastic (*Nf1*^flox/flox^) optic nerve (n.s., not significant for DESeq2 test, p-values adjusted using Benjamini-Hochberg).

Finally, to determine whether differential *ETV5* expression is also a feature of human pediatric low-grade gliomas (pilocytic astrocytoma; PA), we leveraged the only two human RNA microarray datasets that contained reference non-neoplastic tissue for comparison (GSE42656 and GSE12907). We chose these datasets for two major reasons: First, there are no currently-available datasets with RNA expression data on NF1-associated optic glioma and normal optic nerve, since these tumors are rarely biopsied as part of routine medical care. Second, while the genetic etiology of sporadic and NF1-associated PA are distinct, they both result in activation of the same growth control pathways [3, 35–39] and are histologically similar [40].

**Fig 6:**
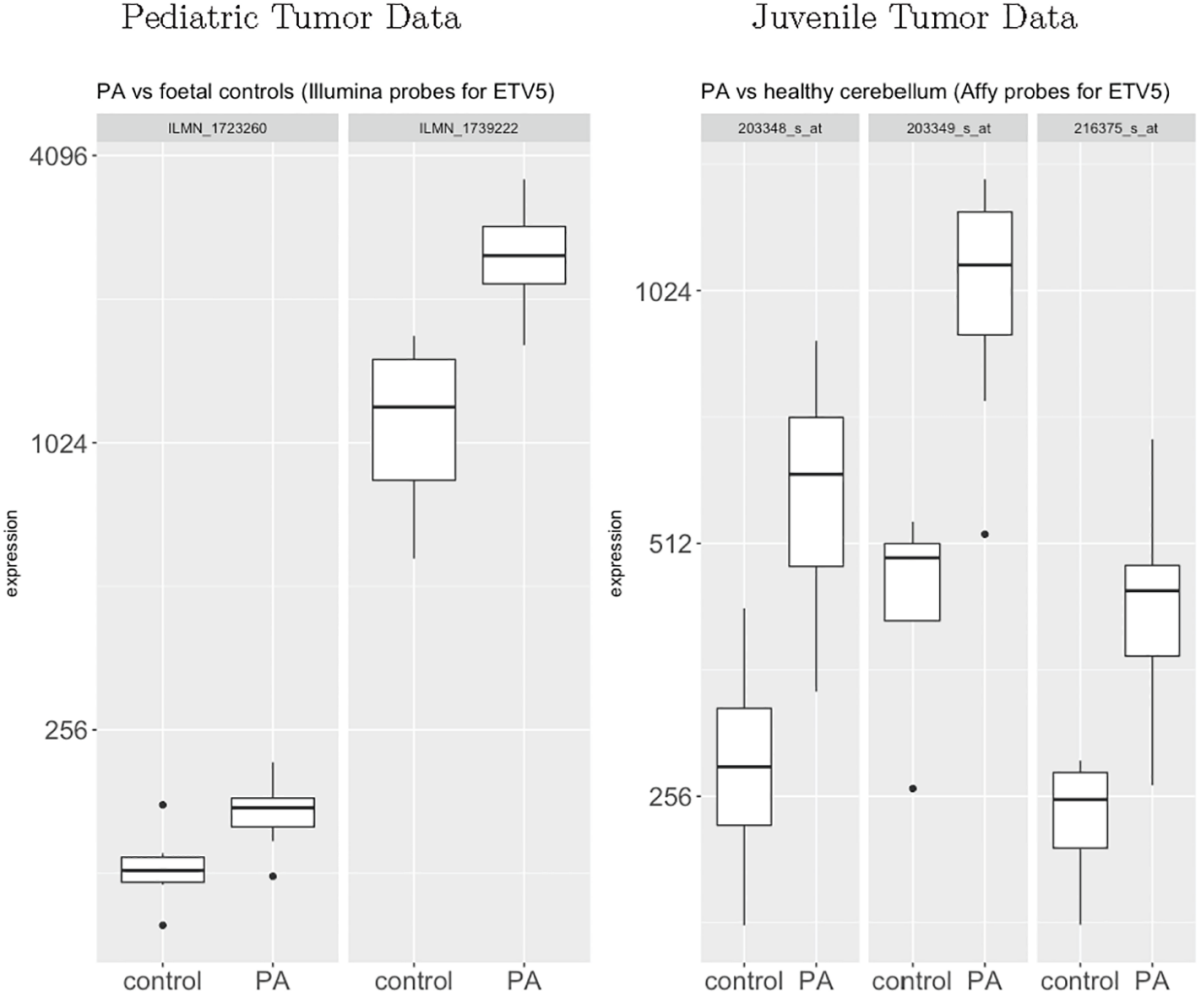
Differential expression of *ETV5* in human pilocytic astrocytomas. *ETV5* is differentially expressed in the tumor groups from both datasets. Using the pediatric pilocytic astrocytoma data, both of the two Illumina probes for *ETV5* are significantly differentially expressed (p < 0.0001 for both probes). Using the juvenile pilocytic astrocytoma data, all three of the Affymetrix probes are significantly differentially expressed (p = 0.001, p = 0.00005, p = 0.00003 for the three probes). Vertical axis is on a log2-scale.

**Fig 7:**
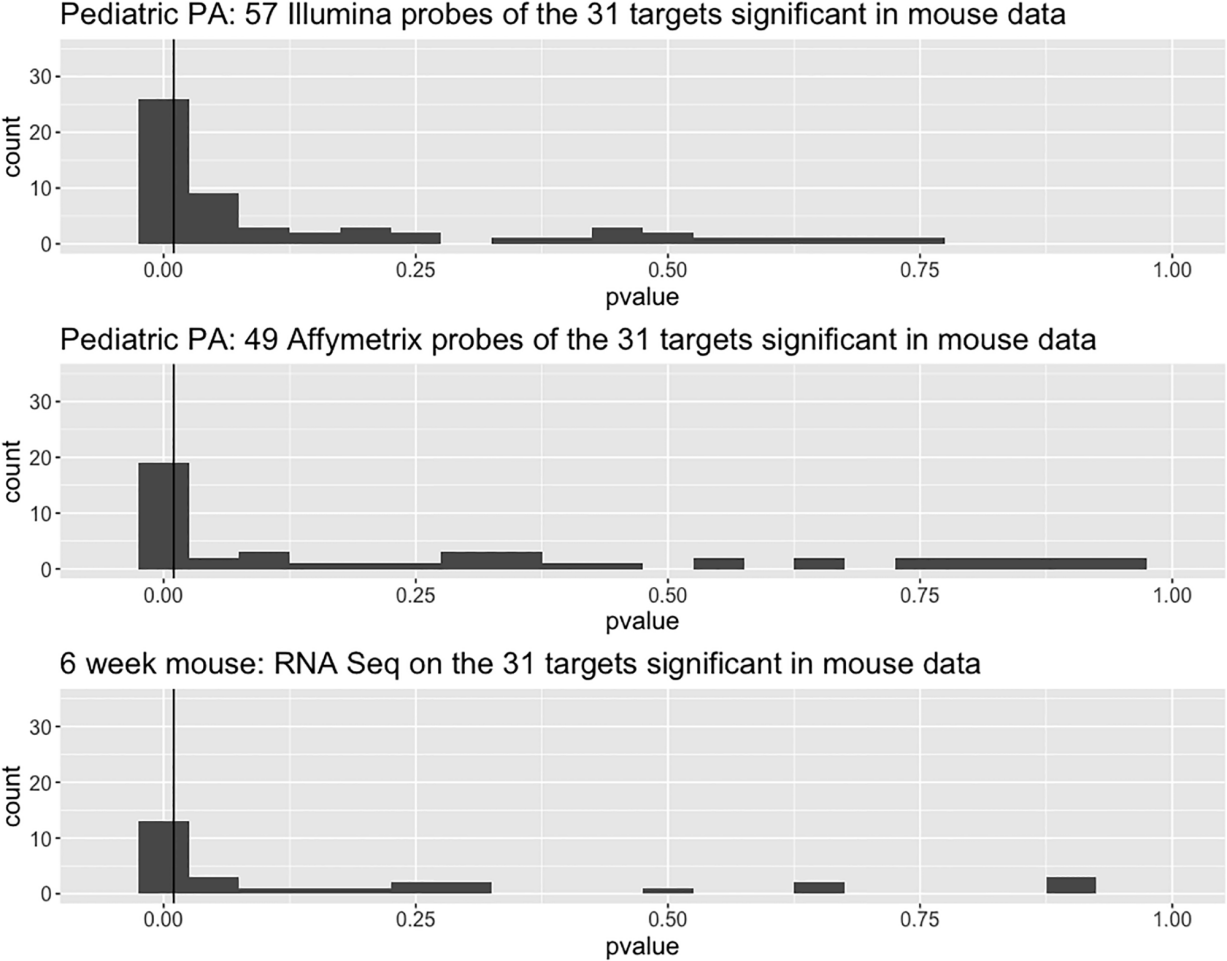
Differential expression of *ETV5* network genes in human pilocytic astrocytoma. Histograms of (unadjusted) p-values for differential expression of *ETV5* target genes in two human data sets; vertical lines are drawn at p = 0.05. In the GSE42656 dataset, 56.1% of all probes and 64.5% of the target genes are significantly differentially expressed (top panel). In the GSE12907 dataset, 40.8% of all probes and 38.7% of the target genes were significantly differentially expressed (middle panel). In the 6-week-old murine OPG-1 data set, 48.3% of the target genes were significantly differentially expressed (lower panel).

In both the pediatric and the juvenile pilocytic astrocytoma datasets, *ETV5* was differentially expressed in the tumor groups relative to the non-neoplastic samples (Fig 6), similar to that observed in the mouse low-grade gliomas. Moreover, using the thirty-one *Etv5* target genes that were differentially expressed in the murine tumors (Table 2), a large fraction of the probes for those targets were also differentially expressed (unadjusted p-values) in the two human datasets (56.1% in the pediatric pilocytic astrocytoma dataset and 40.8% of the probes in the juvenile pilocytic astrocytoma dataset; Fig 7).

## Discussion

Leveraging the betweenness network analysis and RNA-sequencing of murine optic glioma tissues, we discovered *Etv5* and *Etv5*-associated genes as a differentially-regulated transcriptional network in low-grade brain tumors relative to non-neoplastic brain tissue. We further validated the differential expression of *Etv5* and its target genes in several other murine models of *Nf1* optic glioma, as well as in human pilocytic astrocytomas. This type of network analysis is extremely useful in identifying potential network regulators unique to the neoplastic state, since multiple differentially-expressed genes were identified in tumor versus normal healthy tissue. In addition, the deployment of multiple computational and statistical analysis strategies, as well as biological validation across species and time further strengthen the idea that *Etv5* may be a central control element in establishing and/or maintaining low-grade gliomas. Moreover, the identification of *Etv5* and its network as a tissue-level signature is supported by the lack of differential *Etv5* expression in isolated *Nf1*-deficient astrocytes or *Nf1*-mutant microglia, which are the major neoplastic and non-neoplastic cells in the optic glioma ecosystem.

ETV5 is a transcription factor that belongs to the conserved E26 transformation-specific family of transcription factors. Relevant to our study, ETV5 levels are elevated in many tumors, including glioma [41–43]. As such, ETV5 has been hypothesized to function downstream of RAS [44], which is consistent with the major role of the *Nf1* protein (neurofibromin) as a negative regulator of RAS in the brain [3, 19]. In addition, ETV5 function is required to sustain *Nf1*-deficient high-grade glioma growth [43], but had not been previously implicated in low-grade gliomas. Since ETV5 expression is not a hallmark of any particular neoplastic cell type in the low-grade gliomas, it is most likely that the network established reflects aberrant RAS activation in numerous cell types in the tumor. Future mechanistic studies will be required to address the relationships between NF1/RAS/MEK/ERK pathway function, ETV5 network regulation, and NF1-associated low-grade glioma formation or maintenance.

Taken together, we present a proof-of-concept study that establishes betweenness network analysis as a valuable tool for identifying central genes unique to the tumor ecosystem. While this approach nicely illustrates the value of computational and bioinformatic approaches for characterizing the neoplastic state relative to its non-neoplastic counterpart, further investigation is required to understand the mechanistic role of Etv5 in glioma formation and maintenance. Based on these findings, we recommend the use of network analysis for similar expression studies designed to differentiate various tumor states.

## Supporting information

S1 Fig Comparison of closeness measures in the normal and tumor networks. Filled (red) circles indicate genes whose betweenness measure is at least 1.1 times as large in the tumor network as in the normal network and either a tumor betweenness or normal betweenness value greater than 1e6 (identified in the body of the manuscript). These genes are listed in Table 1, and shown in pink in Fig 4. Note that the closeness metric does not differentiate the tumor and normal networks as well as betweenness (see Fig 3).
(TIFF)

S2 Fig *Etv5* expression in 6-week-old optic glioma-bearing mice. *Etv5* RNA expression is higher in 6-week-old optic glioma-bearing mice relative to control non-neoplastic optic nerves (three samples for each experimental group; p = 0.0113). Note that there is no overlap in the expression of ETV5 for the control versus optic glioma-bearing nerves.
(TIFF)

S3 Fig *Nf1*-independent regulation of *Etv5* expression in primary astrocytes. No differences in *Etv5* mRNA expression were observed between wild-type and *Nf1*-deficient astrocytes, as assessed by quantitative real-time RT-PCR. Three independently-generated pairs of wild-type and *Nf1*-/- primary brainstem astrocytes were generated, and maintained as previously described [1–3].
(TIFF)

S1 Table Primers used for real-time quantitative PCR. (DOCX)

S2 Table The gene regulatory network used as a reference network. (XLSX)

S1 References References associated with the supplementary information. (DOCX)
