# Supplementary figures and images for: Graph complexity analysis identifies an ETV5 tumor-specific network in human and murine low-grade glioma
Source: PLoS One. 2018 May 22;13(5):e0190001. doi: 10.1371/journal.pone.0190001 (PMC5963759; doi:10.1371/journal.pone.0190001)

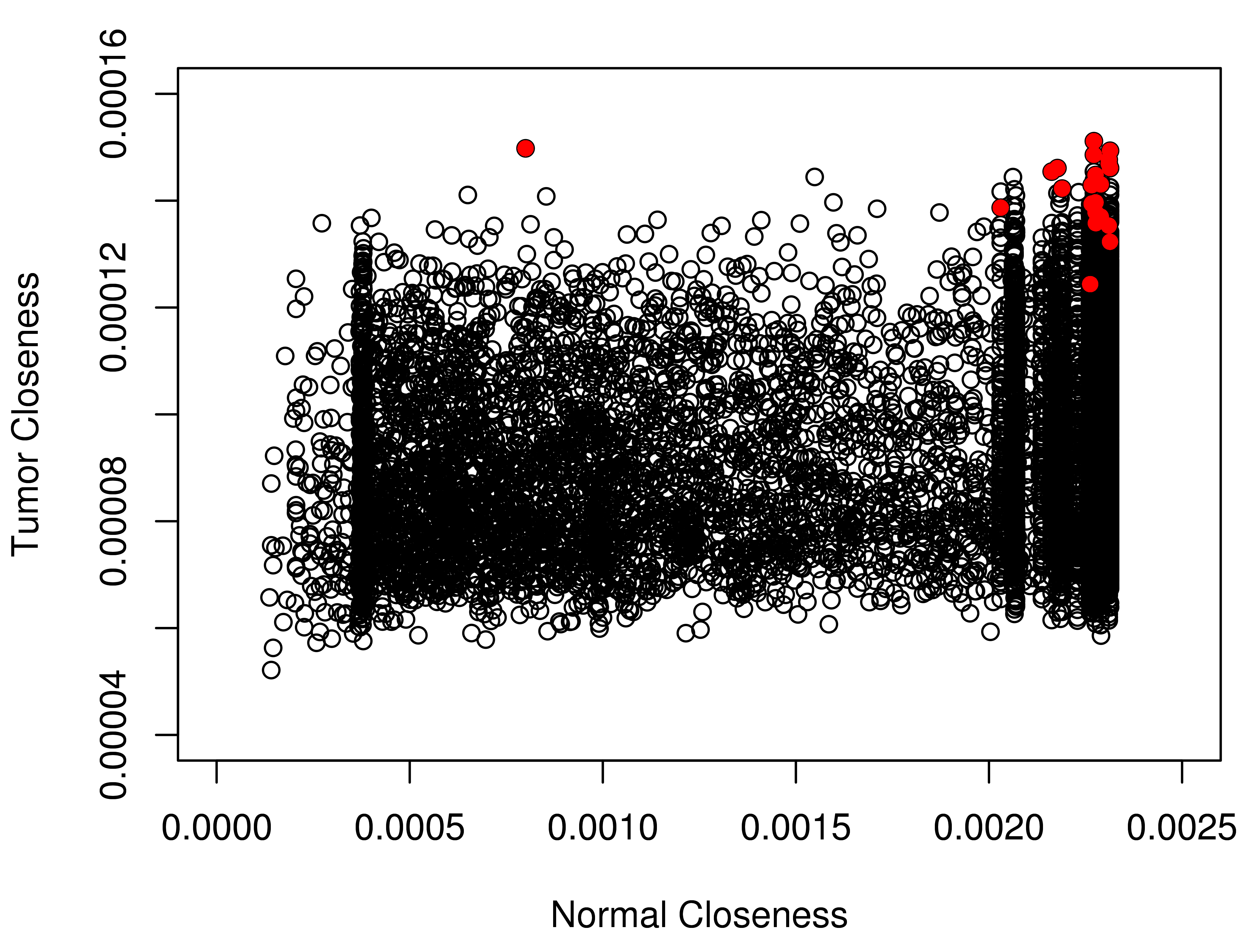

Supplement: S1 Fig — Filled (red) circles indicate genes whose betweenness measure is at least 1.1 times as large in the tumor network as in the normal network and either a tumor betweenness or normal betweenness value greater than 1e6 (identified in the body of the manuscript). These genes are listed in Table 1, and shown in pink in Fig 4. Note that the closeness metric does not differentiate the tumor and normal networks as well as betweenness (see Fig 3). (TIFF) [file pone.0190001.s001.tiff]

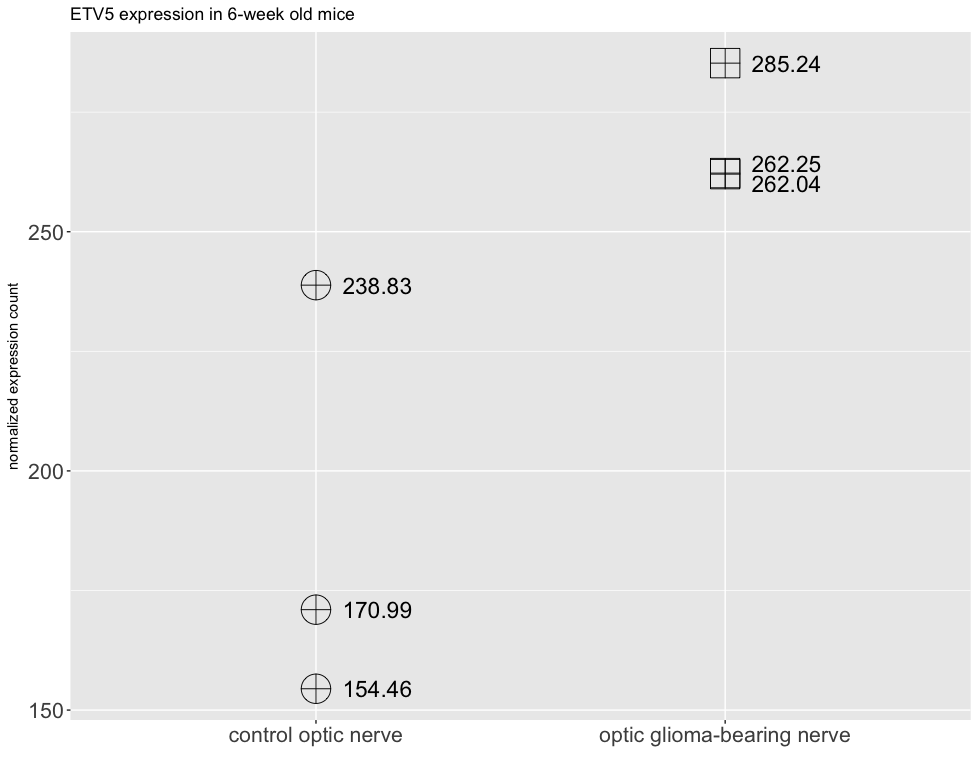

Supplement: S2 Fig — Etv5 RNA expression is higher in 6-week-old optic glioma-bearing mice relative to control non-neoplastic optic nerves (three samples for each experimental group; p = 0.0113). Note that there is no overlap in the expression of ETV5 for the control versus optic glioma-bearing nerves. (TIFF) [file pone.0190001.s002.tiff]

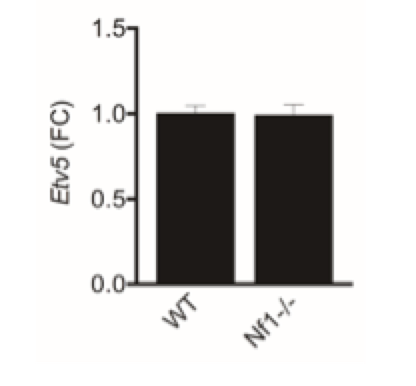

Supplement: S3 Fig — No differences in Etv5 mRNA expression were observed between wild-type and Nf1-deficient astrocytes, as assessed by quantitative real-time RT-PCR. Three independently-generated pairs of wild-type and Nf1-/- primary brainstem astrocytes were generated, and maintained as previously described [1–3]. (TIFF) [file pone.0190001.s003.tiff]
