# Supplementary material for: Graph complexity analysis identifies an ETV5 tumor-specific network in human and murine low-grade glioma
Source: PLoS One. 2018 May 22;13(5):e0190001. doi: 10.1371/journal.pone.0190001 (PMC5963759; doi:10.1371/journal.pone.0190001)
Supplement: S1 Table — (DOCX) [file pone.0190001.s004.docx]

**S1 Table. Primers used for real-time quantitative PCR.**

| **Genes** | **Forward primer** | **Reverse primer** |
| --- | --- | --- |
| *Etv5* | cgcaagtatcatcaaagtacagc | cctaactgccagtcatcctac |
| *Gldc* | tctggctgatacggagtgta | ctggaggtcgtatattggcat |
| *Spry4* | ccaggatatcacccaccattg | tgtgctgctgctgctc |
| *Fabp5* | tgaaagagctaggagtaggactg | ctctcggttttgaccgtgatg |
| *Pcdhgc3* | cttgagagaaacgccagtcag | gcgaagttgtgatcctgtgt |
| *Spry2* | cctctgtccaggtccatcagcactgtcagc | gcagcagcaggcccgtgggagaag |
| *Shc3* | atctccctcttcttcagtcca | ctccggttcaaacagtatttgc |
| *Spred1* | tgatcgaggcattcgaagagct | ggtaacaactgtctcttgctgg |
| *Nlgn3* | gttcacagcccagctagatag | catcctgtgtcagtctccttac |
| *Dusp6* | ggaagtgtgtgtatgtgtgcgagt | ccttcctctcaacctgttgca |
| *Lrp4* | ggcaaaaagcaggaacttgt | tctacccagtggccagaact |
| *Rsbn1l* | gctctcgtcctgtcttcaaac | ctgatatgccaaagtcaccct |
| *Socs2* | ggttgccggaggaacagtc | gagcctcttttaatttctctttggc |
