# Supplementary material for: Graph complexity analysis identifies an ETV5 tumor-specific network in human and murine low-grade glioma
Source: PLoS One. 2018 May 22;13(5):e0190001. doi: 10.1371/journal.pone.0190001 (PMC5963759; doi:10.1371/journal.pone.0190001)
Supplement: S1 References — (DOCX) [file pone.0190001.s006.docx]

**Supplementary References**

1. Solga AC, Pong WW, Kim KY et al. RNA Sequencing of Tumor-Associated Microglia Reveals Ccl5 as a Stromal Chemokine Critical for Neurofibromatosis-1 Glioma Growth, Neoplasia 2015;17:776-788.

2. Dasgupta B, Yi Y, Chen DY et al. Proteomic analysis reveals hyperactivation of the mammalian target of rapamycin pathway in neurofibromatosis 1-associated human and mouse brain tumors, Cancer Res 2005;65:2755-2760.

3. Sandsmark DK, Zhang H, Hegedus B et al. Nucleophosmin mediates mammalian target of rapamycin-dependent actin cytoskeleton dynamics and proliferation in neurofibromin-deficient astrocytes, Cancer Res 2007;67:4790-4799.
